# The critical issue of using lead for sustainable massive production of perovskite solar cells: a review of relevant literature

**DOI:** 10.12688/openreseurope.13428.2

**Published:** 2021-10-28

**Authors:** Simone Maranghi, Maria Laura Parisi, Riccardo Basosi, Adalgisa Sinicropi

**Affiliations:** 1 Department of Biotechnology, Chemistry and Pharmacy, R²ES Lab, University of Siena, Via A. Moro 2, Siena, 53100, Italy; 2 Center for Colloid and Surface Science (CSGI), Via della Lastruccia 3, Firenze, 50019, Italy; 3 Institute for the Chemistry of OrganoMetallic Compounds (CNR-ICCOM), Italian National Council for Research, Via Madonna del Piano 10, Firenze, 50019, Italy

**Keywords:** Perovskite Solar Cells, Environmental Assessment, Toxicity, Life Cycle Assessment, Metals, Lead, Sustainability, Photovoltaics

## Abstract

This work aims to review the most significant studies dealing with the environmental issues of the use of lead in perovskite solar cells (PSCs). A careful discussion and rationalization of the environmental and human health toxicity impacts, evaluated by life cycle assessment and risk assessment studies, is presented. The results of this analysis are prospectively related to the possible future massive production of PSC technology.

## Introduction

As part of the European Green Deal, the European Union (EU) has set the ambitious goal of reducing 55% of its greenhouse gas (GHG) emissions by 2030 and becoming the first continent in the world to be completely climate neutral by 2050 ^1, 2^. To achieve this challenging goal, significant changes will be required in the energy mix of most of the EU countries to reduce dependency on fossil fuels and their consequent GHG emissions. The two other related objectives, namely increasing energy efficiency to 36% and increasing renewable energy share to 38.5%, will be essential to achieve the target defined above. Renewable energy sources are those that renew themselves naturally at rates that are equivalent or higher than the rates of their use, such as solar energy, wind energy, hydropower, marine (tide, wave, ocean) and geothermal energy. They prevalently contribute to electric power and minimally to thermal request. In sharp contrast with all the other fuels, renewables have already shown their resilience to the coronavirus pandemic crisis ^3^. The share of renewables in the energy use of the 27 EU member states was approximately 19.7% at the end of 2019, very close to the 20% target originally established for 2020 ^4, 5^.

On the other hand, the share of renewables in the worldwide electricity supply reached 27% in 2019 ^6^, 7% below the EU for the same year (i.e., 34%) ^4^, which shows that Europe is indeed ahead in the energy transition. Although technologies exploiting renewable energy sources are not always intrinsically ecofriendly, renewable energy is unquestionably important to ensure a sustainable society, in which both citizens and industries can benefit and develop while respecting the replenishing rate of natural resources. In this regard, the 7 ^th^ Sustainable Development Goal (SDG) defined by the United Nations (UN) for 2030 is “Ensure access to affordable, reliable, sustainable and modern energy for all”, stating a clear goal of increasing the share of renewable energy in the total energy usage ^7, 8^. Among the renewables, solar energy is especially important, given the expected increase as part of the decarbonization process, for the energy mix both in EU and in the world. Novel photovoltaic (PV) technologies will play a crucial role in this process, and one of the most promising emerging PV technologies to be recently developed is hybrid halide perovskite solar cells (PSCs).

PSCs have enormously advanced the research and development of innovative PV technologies in the last decade. The power conversion efficiency (PCE) of the cells overcame the record value year by year, reaching 25.5% for single-junction PSC, 24.2% for tandem configuration coupling of the PSC technology with copper indium gallium selenide (PSC/CIGS), 29.5% for the PSC/silicon tandem ^9^, and 17.9% for the perovskite solar module (PSM) ^10^. The extremely high PCE, together with the availability of cell and module configurations, and the low cost of most raw materials and manufacturing techniques, were the pillars of research and development of PSC technology looking for industrialization and high competitivity on the PV market.

However, some not negligible drawbacks need to be overcome to allow PSCs to take the decisive step towards commercialization and thus enter the PV market. The long-term stability and rapid degradation of some components ^11^, the choice of suitable materials and manufacturing procedures for massive industrial scale-up ^12^, and environmental sustainability ^13^ are still open issues that companies and the scientific community are trying to address.

Concerning environmental sustainability, several researchers pointed out the problem of lead (Pb), employed in the crystal configuration of the best performing PSCs. Pb is highly toxic for humans and ecosystems and, if absorbed by living organisms, it negatively affects many internal organs, including the brain, and can bioaccumulate within tissues ^14^.

What makes it extremely dangerous for living organisms’ health is the high mobility and great diffusion potential in the environment that the Pb-containing compounds have. This is due to Pb’s chemical-physical characteristics and its widespread usage until the recent past ^14^. For these reasons, the World Health Organization states that there is no safe level of Pb exposure ^15^, and throughout the RoHS Recast (RoHS2) Directive ^16^, the European Union is pushing for its removal and substitution from a range of electrical and electronic equipment. However, due to its particular characteristics and the scarcity of suitable substitute materials, Pb is still far from being replaced ^17^. Regarding PV applications, although the RoHS2 Directive excludes solar panels from the restrictions (unless they are building or product integrated), as a precaution PSCs should respect 0.1% Pb content as the maximum concentration value tolerated by weight in “homogeneous material”. In this context, the ambiguity of the definition of “homogeneous material” represents a critical point, especially for a perovskite-based device that is characterized by several nano or microlayers made of different materials stacked on top of each other ^16, 18^.

Thus, the toxicity of Pb is one of the most relevant issues to address for safeguarding the environmental sustainability of future industrial production of PSCs. So far, many researchers have investigated the potential toxicological risks caused by the leakage of Pb during the life cycle of PSCs and modules ^19– 24^. At the same time, life cycle assessment (LCA) methodology has been applied to several PSC configurations to evaluate the eco-profiles of the technology ^25– 39^ and understand better which could be the relevant environmental hotspots along the whole PSC life cycle in addition to those related to the use of Pb-based compounds.

Therefore, the concerns regarding the high toxicity of Pb-based compound used in PSCs, and the possible mass production limitations related to the current legislation, are still open issues for which a widespread consensus among the scientific community and manufacturers has not been reached yet ^17^.

This work aims to review all aspects connected with the sustainability and environmental assessment of Pb employed in PSCs to highlight the significant issues that should be taken into account to guarantee safe industrial development and massive exploitation of this technology. To do so, we first describe the physico-chemical properties of Pb, why it is pivotal for the PSC development and what are the potential risks for humans and the environment. Afterward, we outline the major outcomes of environmental analysis studies (LCA, toxicological and risk assessment) on PSCs, focusing on the toxicity-related environmental impacts. Next, we present the most recent mitigation and encapsulation techniques developed and published in last years, and finally, we outline the main end-of-life concerns and potential future recycling techniques.

## Pb in PSCs

**Figure 1.**
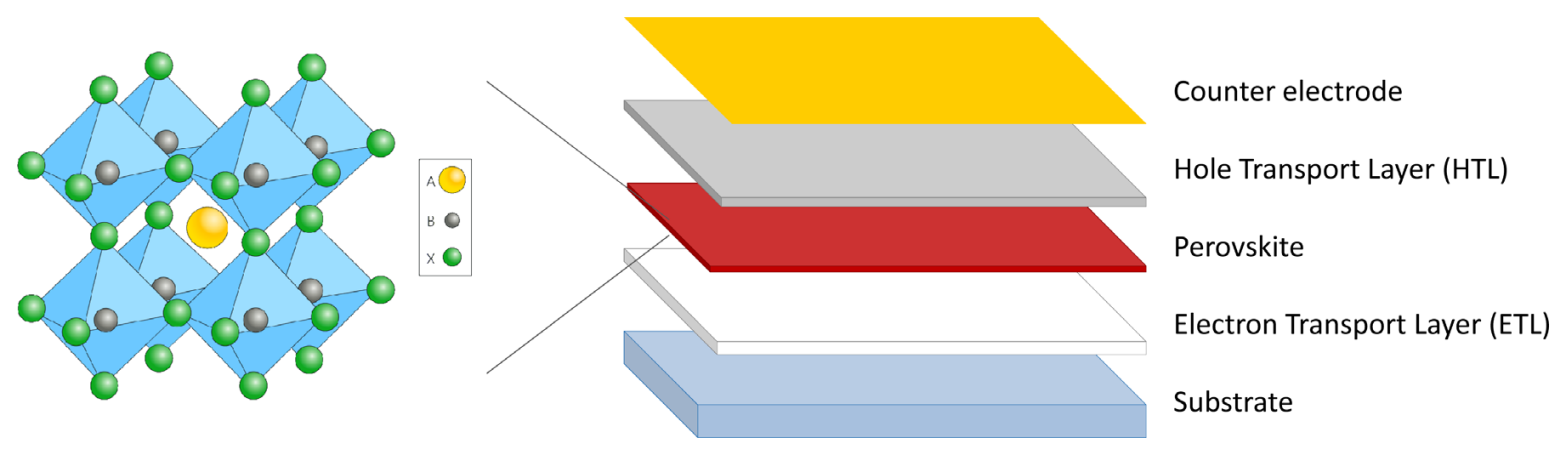
Crystal structure of perovskite (on the left) and conventional configuration of a perovskite solar cell (on the right).

Conventional Pb-based perovskites show a distinctive crystal structure featuring the ABX _3_ pattern where A is an organic or inorganic cation (usually methylammonium, formamidinium or cesium), B is Pb, and X is a halide (usually iodine or bromine). Perovskite is the PSCs’ photoreactive compound, and it cooperates with several other materials and compounds in the cell to convert light into electricity ( Figure 1).

The peculiarities of this crystal structure are the main factor that leads to the astonishing photoconversion efficiency PSCs have been showing so far. They are related to several optoelectronic parameters such as bandgap, absorption coefficient, carrier diffusion lengths, trap density, shallow defects and exciton binding energy ^40^. For this reason, despite several attempts to replace Pb with other metals ^40– 42^ such as tin (Sn), germanium (Ge), bismuth (Bi), antimony (Sb) or indium (In), no viable, effective and compelling alternative has been found yet.

From an operational perspective, the highest efficiency recorded for a Pb-free PSC is 10.9%, reached with a Sn-based perovskite ^43, 44^. Sn has a similar electronic configuration to Pb, and it seems to be the most promising candidate to replace Pb in perovskite for PV applications. However, low PCE and concerns about the environmental impact of Sn-based perovskite ^21, 24^ have slowed down the development of Sn-based PSCs so far. In this context, several researchers are addressing their attempts and effort in the direction of investigating and examining in depth the potential risk of using Pb in PSCs.

### Toxicology issues

Hailgnaw *et al.* analyzed Pb leakage in case of damage and exposure to rain of one Pb-based PSM ^19^. Due to the high mobility and the potential solubility in water of Pb-based compounds originating from the perovskite (including also lead iodide, PbI _2_, that, despite its moderate solubility in water, can release not-negligible quantities of the metal over time), they showed almost all the Pb leaked from the module, generating relevant pollution in all the environmental compartments (i.e., water, soil, and air) in the surroundings of the PSM installation. The authors stated that the concentration of Pb in the soil could increase by about 70 ppm, where the typical range of concentration for natural uncontaminated soil is <10–30 ppm, and 50–200 ppm in urban areas ^19^.

Babayigit *et al.* assessed the toxicity of the PbI _2_ compound by measuring the statistically derived dose descriptors LC50 (i.e., lethal concentration for 50% of the population) and EC50 (the effect concentration for 50% of the population) in a population of zebrafish ^21, 22^. The authors revealed that both death and adverse impacts on the organisms occurred at a low concentration of PbI _2_ in water. The synergistic effect of the exposure to Pb-based compounds and the decrease in pH caused by the formation of hydrogen iodide (HI) was crucial in obtaining these results.

Another valuable contribution for evaluating the toxicity of Pb-based compounds comes from Li *et al.*, which experimented with the uptake of Pb-based perovskite in mint plants ^23^. The authors measured the amount of Pb in roots, stems, and leaves of plants grown in contaminated soil. They found an increase of 10% in the amount of Pb in soil caused an increase of Pb content in plants higher than 100%. Furthermore, the presence of organic cations of the perovskite (methylammonium in this case) has a remarkable effect on the uptake.

Benmessaud *et al.* evaluated the cytotoxicity of Pb-based perovskite contamination in several human cells, highlighting the onset of different damaging effects, from a reduction in cell reproduction to cell death ^45^.

### Comparison with other sources of Pb emissions

Despite the need for a deepened, detailed, and all-encompassing investigation of the toxicity-related issues, the potential risk and damage to humans and ecosystems associated with pollution by Pb-based compounds seems to be sufficiently documented. Besides that, the appraisal of environmental implications connected to large production of PSCs with a prospective approach can help to determine the eco-profiles of electricity produced by this technology, thus being an essential contribution to appropriate assessment of the whole issue.

Fabini attempted to quantify the Pb content in PSCs that would be required to supply the electricity mix in the US, comparing this value with the amounts of Pb-based compounds emitted by other sectors ^20^. Results are reported in Table 1.

**Table 1.** United States (US) lead (Pb) emission sources and hypothetical Pb content in perovskite solar cells (PSCs) to supply the entire US electricity sector. The emission values have been detected in the years reported in brackets, but they are still relevant in 2021. Data is taken from Fabini, 2015 ^20^.

Lead Emission Source	Total Value (tonnes/year)	Compartment
Automotive fuel (1973)	2∙10 ^5^	Airborne emissions
Aviation fuel (2011)	4.40∙10 ^2^	
Metals processing (2011)	1.20∙10 ^2^	
Electricity generation (2011)	3.50∙10 ^1^	
Coal ash, blackwater (2011)	5.90∙10 ^3^ - 9.30∙10 ^4^	Liquid & solid content
Electronic solder (2012)	6.20∙10 ^3^	
PSCs to supply electricity	1.60∙10 ^2^	Solid content

Apart from Pb emissions generated by automotive fuels before tetraethyl lead (Pb(C _2_H _5_) _4_) was removed as an additive in gasoline, all other Pb emissions sources are still relevant and present in 2021. Given that only a small percentage of Pb employed in PSCs could be directly emitted into the environment, the analysis shows that potential pollution caused by future large-scale production of PSCs may be lower than or analogous to other current Pb emission sources. In this framework, the environmental compartment into which emissions flow considerably affects the behavior and potential toxicity of Pb-based compounds. Direct emission into the air could lead to diffused pollution, also affecting other environmental compartments, such as soil or water, and thus, indirectly, food. It is well known that treatment and disposal of coal ash and blackwater are hazardous, and could lead to environmental disaster ^20^. In contrast, Pb content in perovskite is in a solid form and, if adequately encapsulated, its mobility could be adequately limited, and the consequent emission into the environment could be very low.

Hauck *et al.* performed a LCA to analyze the prospective contribution that large-scale production and installation of PSCs could make to the transition toward an energy system based on renewable sources ^25^. In their work, the electricity produced by PSCs/Si modules in a tandem configuration is compared with the electricity produced by the average European electricity mix. Despite some significant assumptions and approximations, the authors concluded that a substantial reduction of Pb-based compound and GHG emissions could be achieved by replacing conventional energy production technologies with PSCs.

Billen *et al.* followed a similar approach by applying LCA to calculate the environmental impact and toxicity potential of electricity produced by Pb-based perovskite PV and comparing them to the US electricity mix eco-profile ^26^. The major outcomes show that the environmental footprint of the kWh generated by PSCs could decrease Pb emissions by a factor of 2–4. The authors state that the potential Pb emissions related to PSCs could be marginal compared to those caused by conventional energy production technologies. In this context, the emission of toxic compounds that could potentially occur during the manufacturing phase, together with the unlikely emission of the whole Pb content during end-of-life, could be offset in only two years of operation.

### Availability, viable alternatives and recyclability

**Figure 2.**
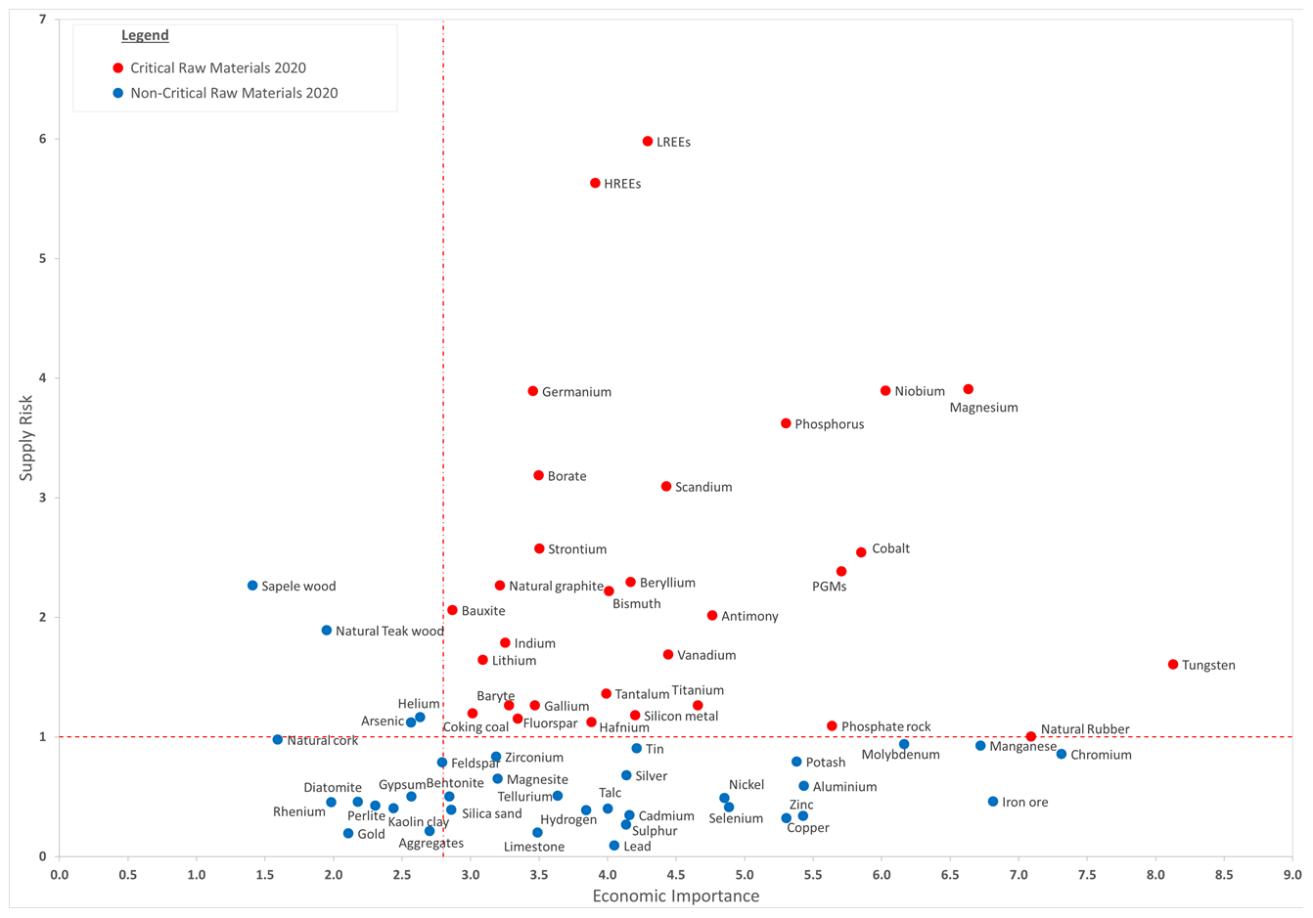
Critical Raw Materials list 2020 (Reproduced from “European Commission, Study on the EU’s list of Critical Raw Materials (2020)” ^70^).

The availability of raw materials and metals is one of the main issues to address when evaluating the sustainability of energy-generating technologies and innovative devices for the exploitation of renewable energy sources ^46^. Among all the metals that could be employed in PSC devices, Pb shows some advantages, one of the most convincing being the availability of its natural reservoirs ^47– 49^ as shown in Figure 2, which is taken from the European Commission Raw Materials Information System (RMIS) ^50^.

The critical raw material (CRM) diagram in Figure 2 points out that Pb ores have good availability, especially compared with other rare metals. In addition to this, due to the high recycling rate, a significant source of secondary Pb comes from recycling procedures ^48^. In 2016, in the US, the amount of recycled Pb was more than 80%, while in Europe it was around 60% ^18, 24^.

The significant recycling rate of Pb allows for hypothetical end-of-life management of PSCs that could limit and hopefully totally ward off the disposal of Pb-based electronic waste in landfills ^51^. As has already been done for conventional Si-based ^52^ and CdTe ^53^ panels, a strategic plan for PSC systematic recycling engaging producers, sellers, consumers, and electronic waste recovery companies needs to be implemented as soon as the commercialization of PSCs starts. In this regard, several authors have already explored and investigated the feasibility of the PSC recycling process ^54, 55^, highlighting the high recycling rate of Pb ^56^ and the potential recovery of most to all of the PSC components ^57– 64^. Although these processes have been demonstrated at a laboratory scale and need to be scaled-up, the prospect of manufacturing PSCs with recovered materials, avoiding efficiency losses, has been already documented ^51, 65– 68^.

Analyzing the possible replacement of Pb with other metals, some critical issues need to be addressed. As reported above, metals identified and tested as a viable alternative for metal-based perovskite in PSCs are Sn, Ge, Bi, Sb and In ^40^. Figure 2 shows that most of these metals’ availability is lower than Pb. These elements are scarce or even very rare, and they are employed for other uses. Roughly comparing annual production, it can be noted that the total amount of some alternative metals produced would not be sufficient to be employed in PV device manufacturing ^24, 49, 69^.

The potential replacement elements should fulfill some crucial performance standards to be competitive with Pb. In addition to the already mentioned natural availability and ease of recycling, alternative metals should form a stable perovskite structure that exhibits outstanding optoelectronic properties and excellent PCE, they should be characterized by a low-cost supply chain and manufacturing procedure, and they should satisfy some commercial requirements, such as long-term stability and scalability ^40^.

Moreover, together with these stringent criteria, issues related to the whole life cycle of metals and their relative environmental sustainability must be considered. Nuss *et al.* performed a cradle-to-gate LCA of metals, illustrating the numerous interconnections among the manufacturing procedures and assessing the related environmental burdens ^71^. One of the major outcomes of the study deals with the common sources and extraction procedures characterizing some elements. This aspect substantially influences the market availability and cost of some metals, whose production is commercially attractive only as a by-product of other metals ^24^. From the environmental assessment perspective, among all metals, Pb displays one of the lowest impacts for all the environmental categories and indicators considered by Nuss *et al.* (i.e., GHG emissions, cumulative energy demand, terrestrial acidification, freshwater eutrophication, and human toxicity) ^71^. In addition to this, the authors state that for some metals, the mining and concentration and the subsequent purification and refining steps exhibit approximately similar environmental burdens. These steps are also a major contributor to the environmental profile of the whole metal’s life cycle ^71^. Thus, the impact could be strongly reduced using secondary recycled Pb and implementing a thorough end-of-life strategy for PSC technologies.

## LCA for environmental assessment of Pb in PSCs

The LCA methodology has been extensively applied to assess PSCs manufacturing’s environmental impact and operational phase. Many different configurations of cells and modules employing several raw materials and chemical compounds and requiring various manufacturing procedures and deposition techniques have been analyzed in recent years ^17– 29^. To highlight the main potential environmental hotspots of PSCs, we performed a critical review and in-depth harmonization of LCA studies published (2019) ^36^ in the frame of the H2020 Project “ESPResSo”. The major outcomes and results of the study are consistent with those reported in similar papers published more recently ^27, 29^, and they can be summarized as follows.

The main hotspots in terms of materials employed for PSCs production are gold (used as back contact), the conductive solar glass, and the electron transport material (ETM) due to raw materials consumed during the synthesis. Regarding the manufacturing procedures, the back contact deposition, the ETM deposition and the glass substrate preparation show remarkably high environmental impacts due to their direct energy consumption ^36^. Some manufacturing techniques have been found to be better than others for bringing PSCs to the industrial production scale with competitive deposition efficiency (e.g., ink-jet printing, slot-die coating, spray-coating) ^27^, and a global consensus on the replacement of gold as the material for back contact has been achieved ^36^.

Concerning the toxicity issues of PSCs, and the use of Pb-based compounds in particular, nearly all of the studies come to similar conclusions ^17– 29^. From an LCA perspective, the Pb and Pb-based compounds’ burden on the environmental profile of PSCs can be considered substantially negligible. This is due to two main factors: i) the limited environmental impact that the production of Pb ore displays in LCA analysis ^71^, and ii) the exiguous amount of Pb in PSCs and the relative contribution to the global environmental footprint of devices, which is some orders of magnitude lower than those of other materials and processes required in the manufacturing procedure. However, despite the relatively low overall burden, the presence of Pb contributes highly to the toxicity-related categories ^36^. This outcome can be explained through the discussion of the so-called characterization factors (CFs) of metals that are applied in the life cycle impact assessment step as weighting factors to aggregate life cycle emissions into scores for human health and ecosystem health impacts.

**Figure 3.**
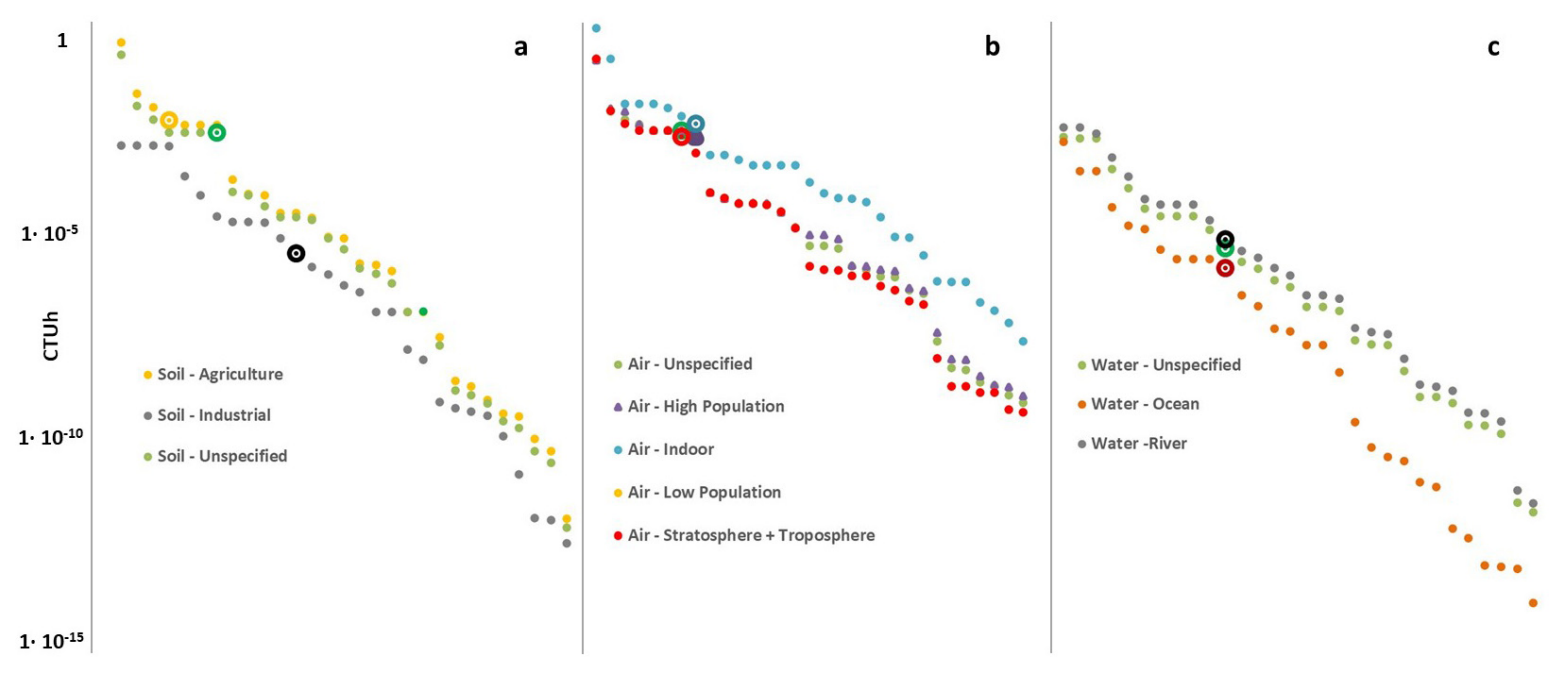
Plot of the toxicity-related characterization factors of heavy metals including lead (larger dots in the chart) for the impact category human toxicity non-cancer effect, relative to the environmental compartment soil ( **a**), air ( **b**) and water ( **c**).Characterization factors are expressed in comparative toxic unit for humans (CTUh). Data is taken from the Environmental Footprint Life Cycle Impact Assessment Method ^72, 76^.

Figure 3a, b and c show the CFs of Pb in comparison with those of other heavy metals Values are derived from the most updated version of the Environmental Footprint (EF) method, which includes the USEtox model ^72^ for the evaluation of the potential toxicity of substances (the most highly recommended ^73, 74^ impact assessment method).

Examining the characterization factors reported in Figure 3a, b and c, it is clear that Pb could generate non-negligible impacts and could be a potentially serious risk if it was emitted into the environment. For example, concerning the human toxicity non-cancer impact category relative to the soil and air compartments, the potential toxicities of Pb results are extremely high, especially if the Pb-based compounds are emitted in agricultural soil or indoor air. Moreover, Pb exhibits high potential toxicity for all air sub-compartments in the same category.

These outcomes agree with and strengthen those reported by studies focusing on toxicity modeling and risk assessment of Pb-compound emissions from PSCs ^19– 24^, suggesting that the evaluation of potential toxicity of Pb-based PSCs could be still an open question. The main risk is underestimating the potential damage of local emissions and danger associated with small-scale pollution during the manufacturing process, use phase and end-of-life management. The point is that since the toxicity of a metal depends on several physico-chemical parameters such as oxidation state, ligands, solubility, morphologies, characteristics of the environment, and many others, the effect on biological systems should not be assumed or taken for granted ^75^. Therefore, there is a further need for reliable measurements and empirical tests to improve knowledge of the toxicity of Pb-based PSCs.

For this reason, it is important to specify that the application of LCA for the evaluation of toxicity of specific metal-based compounds might not be exhaustive and might lead to results with relatively high uncertainty. This is due to the inherent uncertainty of the USEtox model related to the lack of some physico-chemical parameters used to model the fate, exposure, and the potential toxicity of many metal-based compounds ^72^.

## Encapsulation as a viable solution to facilitate commercialization

The most promising solution to mitigate the risks associated with Pb emission and leakage during the use and end-of-life phases is the physical encapsulation of PSCs and PSMs. Encapsulation is a standard procedure that uses different materials to cover and protect the laminated module from external agents. This procedure allows enhancement of the module’s stability by limiting the oxidation and degradation of materials, and recent development demonstrates that physical encapsulation could also reduce Pb-based compounds’ emission by sequestrating most of the Pb in the PSCs ^77– 79^.

In recent studies, the encapsulated modules have been subjected to various stress tests, such as: i) mechanical shattering followed by water soaking ^77^; ii) fire simulation ^78^; and iii) mechanical damage followed by simulated rainfall ^79^, and, definitively, all studies came to similar conclusions.

The limitation of Pb leakage from shattered and soaked PSCs in water was substantial, exhibiting percentages of sequestration efficiency higher than 96% of the total Pb mass for all test conditions (i.e., different water temperature, pH, and soaking time). In addition to this, the PCE of the PSCs encapsulated with Pb-adsorbing films showed no appreciable differences compared with the non-encapsulated PSCs ^77^.

The results of the fire simulation tests outlined that glass encapsulation of the PSCs could avoid the formation of Pb-based compounds that are soluble in water, while facilitating the formation of Pb-based compounds that dissolved into the softened glass, thus limiting their emission into the surrounding environment. The monitoring of air emissions proved that the maximum simulated value of Pb emissions did not exceed the safety standards set by the European Commission ^78^.

The encapsulation with epoxy resins and the subsequent exposure of PSCs to different weather conditions showed a remarkable reduction in Pb-based compound leakage. The most promising encapsulation method under the most severe rainfall simulation (i.e., acid rain) exhibits a reduction in Pb-leakage of more than two orders of magnitude, also thanks to the high temperatures that the device could tolerate under outdoor conditions ^79^.

Recently Chen and co-authors ^80^ showed the effectiveness of a a low-cost mesoporous sulfonic acid-based lead-adsorbing resin that, when incorporated into PSCs as a scaffold, immobilizes lead ions inside it even if PSCs are exposed to rainwater. Introducing the insulating scaffold not only does not decrease the device efficiency, but also can be scaled up to large-area modules

## Conclusion

The environmental sustainability of PSCs and the issues related to the toxicity of Pb in perovskite have started to be extensively addressed in the scientific literature. Based on the studies published so far, it can be inferred that the topic should be approached from as many perspectives as possible due to the inherent uncertainty associated with the models describing the environmental impact of Pb compounds.

From the toxicological point of view, it seems clear that Pb and Pb-based compounds employed and eventually released by PSCs are extremely dangerous and toxic for living organisms. Strong efforts should be put into the further investigation and characterization of fate, exposure and potential toxicity of all Pb-based compounds that are used and could potentially be emitted during the whole life cycle of PSC devices.

On the contrary, from the LCA perspective, Pb shows a quite limited burden on PSCs’ environmental profile, mainly due to the small impact of the metal’s production process. According to the main outcomes of LCA studies on PSCs, the replacement of some raw materials, the reduction of some chemical compounds’ consumption, the improvement of energy requirements, and the implementation of a safe end-of-life phase are the crucial environmental hotspots that need to be addressed to accomplish industrialization and mass production. However, more detailed and in-depth LCA studies focusing on the life cycle of Pb-based compounds employed are necessary to evaluate the real sustainability of Pb-based PSCs. From this point of view, it would be beneficial to expand on the LCA models to customize the analysis for the specific conditions characterizing the investigated systems. Moreover, it should be considered that it is not the chosen life cycle impact assessment method that gives validity to the result of a LCA analysis, but the accuracy and awareness with which the results obtained are discussed, analyzed and contextualized by the operator.

At the same time, widening the perspective to mitigate risks along the whole value chain, for the technology to have a chance of entering the PV market firmly, manufacturing companies should put effort to i) guarantee the safety of the PSC manufacturing phase work environment, ii) develop reliable encapsulation techniques to prevent Pb leakage during the transportation and use phases, and iii) implement harmless and controlled end-of-life management procedures.

## Data availability

No data are associated with this article.

## References

[1] European Commission: The European Green Deal. European Commission.2019;53. Reference Source

[2] EUCO: European council meeting, December 10-11 2020.Conclusions. Reference Source

[3] IEA: Renewables 2020 Analysis and forecast to 2025.2020. Reference Source

[4] European Commission: Renewable energy statistics.2020. Reference Source

[5] Eurostat: Share of energy from renewable sources 2020.2020. Reference Source

[6] IEA: Global energy review 2019.2020. Reference Source

[7] IEA: Data and statistics: Renewable share in final energy consumption (SDG 7.2), World 1990-2017 2020.2020. Reference Source

[8] Güney T : Renewable energy, non-renewable energy and sustainable development. *Int J Sust Dev World.* 2019;26(5):389–397. 10.1080/13504509.2019.1595214

[9] NREL: Best Research-Cell Effciencies. Reference Source

[10] NREL: Champion Module Efficiencies. Reference Source

[11] Wang R Mujahid M Duan Y : A Review of Perovskites Solar Cell Stability. *Adv Funct Mater.* 2019;29(47):1808843. 10.1002/adfm.201808843

[12] Rong Y Ming Y Ji W : Toward Industrial-Scale Production of Perovskite Solar Cells: Screen Printing, Slot-Die Coating, and Emerging Techniques. *J Phys Chem Lett.* 2018;9(10):2707–2713. 10.1021/acs.jpclett.8b00912 29738259

[13] Dedecker K Grancini G : Dealing with Lead in Hybrid Perovskite: A Challenge to Tackle for a Bright Future of This Technology? *Adv Energy Mater.* 2020;10(31):2001471. 10.1002/aenm.202001471

[14] Flora G Gupta D Tiwari A : Toxicity of lead: A review with recent updates. *Interdiscip Toxicol.* 2012;5(2):47–58. 23118587 10.2478/v10102-012-0009-2 PMC3485653

[15] World Health Organization (WHO): Lead Poisoning and Health.2019. Reference Source

[16] European Commission: Directive 2011/65/EU of the European Parliament and of the Council of 8 June 2011 on the restriction of the use of certain hazardous substances in electrical and electronic equipment (recast).2011. Reference Source

[17] Goetz KP Taylor AD Hofstetter YJ : Sustainability in Perovskite Solar Cells. *ACS Appl Mater Interfaces.* 2021;13(1):1–17. 10.1021/acsami.0c17269 33372760

[18] Babayigit A Boyen HG Conings B : Environment versus sustainable energy: The case of lead halide perovskite-based solar cells. *MRS Energy & Sustainability.* 2018;5:1–15. 10.1557/mre.2017.17

[19] Hailegnaw B Kirmayer S Edri E : Rain on methylammonium lead iodide based perovskites: Possible environmental effects of perovskite solar cells. *J Phys Chem Lett.* 2015;6(9):1543–1547. 10.1021/acs.jpclett.5b00504 26263309

[20] Fabini D : Quantifying the Potential for Lead Pollution from Halide Perovskite Photovoltaics. *J Phys Chem Lett.* 2015;6(18):3546–3548. 10.1021/acs.jpclett.5b01747 26722721

[21] Babayigit A Thanh DD Ethirajan A : Assessing the toxicity of Pb- and Sn-based perovskite solar cells in model organism *Danio rerio.* *Sci Rep.* 2016;6:18721. 10.1038/srep18721 26759068 PMC4725943

[22] Babayigit A Ethirajan A Muller M : Toxicity of organometal halide perovskite solar cells. *Nat Mater.* 2016;15(3):247–251. 10.1038/nmat4572 26906955

[23] Li J Cao HL Jiao WB : Biological impact of lead from halide perovskites reveals the risk of introducing a safe threshold. *Nat Commun.* 2020;11(1):310. 10.1038/s41467-019-13910-y 31964862 PMC6974608

[24] Schileo G Grancini G : Lead or no lead? Availability, toxicity, sustainability and environmental impact of lead-free perovskite solar cells. *J Mater Chem C.* 2021;9:67–76. 10.1039/D0TC04552G

[25] Hauck M Ligthart T Schaap M : Environmental benefits of reduced electricity use exceed impacts from lead use for perovskite based tandem solar cell. *Renew Energy.* 2017;111:906–913. 10.1016/j.renene.2017.04.044

[26] Billen P Leccisi E Dastidar S : Comparative evaluation of lead emissions and toxicity potential in the life cycle of lead halide perovskite photovoltaics. *Energy.* 2019;166:1089–1096. 10.1016/j.energy.2018.10.141

[27] Leccisi E Fthenakis V : Life-cycle environmental impacts of single-junction and tandem perovskite PVs: a critical review and future perspectives. *Prog Energy.* 2020;2:032002. 10.1088/2516-1083/ab7e84

[28] Parisi ML Maranghi S Sinicropi A : Development of Dye Sensitized Solar Cells: a Life Cycle Perspective for the Environmental and Market Potential Assessment of a Renewable Energy Technology. *International Journal of Heat and Technology.* 2015;31:143–148. Reference Source

[29] Sarialtin H Geyer R Zafer C : Life cycle assessment of hole transport free planar–mesoscopic perovskite solar cells. *J Renew Sustain Ener.* 2020;12(2):023502. 10.1063/1.5129784

[30] Gong J Darling SB You F : Perovskite photovoltaics: life-cycle assessment of energy and environmental impacts. *Energy Environ Sci.* 2015;8(7):1953–1968. 10.1039/C5EE00615E

[31] Celik I Song Z Phillips AB : Life cycle analysis of metals in emerging photovoltaic (PV) technologies: A modeling approach to estimate use phase leaching. *J Clean Prod.* 2018;186:632–639. 10.1016/j.jclepro.2018.03.063

[32] Celik I Song Z Cimaroli AJ : Life Cycle Assessment (LCA) of perovskite PV cells projected from lab to fab. *Sol Energy Mater Sol Cells.* 2016;156:157–169. 10.1016/j.solmat.2016.04.037

[33] Serrano-Lujan L Espinosa N Larsen‐Olsen TT : Tin‐ and lead‐based perovskite solar cells under scrutiny: An environmental perspective. *Adv Energy Mater.* 2015;5(20):1501119. 10.1002/aenm.201501119

[34] Espinosa N Serrano-Luján L Urbina A : Solution and vapour deposited lead perovskite solar cells: Ecotoxicity from a life cycle assessment perspective. *Sol Energy Mater Sol Cells.* 2015;137:303–310. 10.1016/j.solmat.2015.02.013

[35] Zhang J Gao X Deng Y : Comparison of life cycle environmental impacts of different perovskite solar cell systems. *Sol Energy Mater Sol Cells.* 2017;166:9–17. 10.1016/j.solmat.2017.03.008

[36] Maranghi S Parisi ML Basosi R : Environmental profile of the manufacturing process of perovskite photovoltaics: Harmonization of life cycle assessment studies. *Energies.* 2019;12(19):3746. 10.3390/en12193746

[37] Tian X Stranks SD You F : Life cycle energy use and environmental implications of high-performance perovskite tandem solar cells. *Sci Adv.* 2020;6(31):eabb0055. 10.1126/sciadv.abb0055 32789177 PMC7399695

[38] Tian X Stranks SD You F : Life cycle assessment of recycling strategies for perovskite photovoltaic modules. *Nat Sustain.* 2021;4(9):821–829. 10.1038/s41893-021-00737-z

[39] Parisi ML Sinicropi A : Closing the loop for perovskite solar modules. *Nat Sustain.* 2021;4:754–755. 10.1038/s41893-021-00735-1

[40] Zhang Q Hao F Li J : Perovskite solar cells: must lead be replaced - and can it be done? *Sci Technol Adv Mater.* 2018;19(1):425–442. 10.1080/14686996.2018.1460176 29868147 PMC5974705

[41] Ke W Kanatzidis MG : Prospects for low-toxicity lead-free perovskite solar cells. *Nat Commun.* 2019;10(1):965. 10.1038/s41467-019-08918-3 30814499 PMC6393492

[42] Abate A : Perovskite Solar Cells Go Lead Free. *Joule.* 2017;1(4):659–664. 10.1016/j.joule.2017.09.007

[43] Chen M Dong Q Eickemeyer FT : High-Performance Lead-Free Solar Cells Based on Tin-Halide Perovskite Thin Films Functionalized by a Divalent Organic Cation. *ACS Energy Lett.* 2020;5(7):2223–2230. 10.1021/acsenergylett.0c00888

[44] Nasti G Abate A : Tin Halide Perovskite (ASnX _3_) Solar Cells: A Comprehensive Guide toward the Highest Power Conversion Efficiency. *Adv Energy Mater.* 2020;10(13):1902467. 10.1002/aenm.201902467

[45] Benmessaoud IR Mahul-Mellier AL Horváth E : Health hazards of methylammonium lead iodide based perovskites: Cytotoxicity studies. *Toxicol Res (Camb).* 2015;5(2):407–419. 10.1039/c5tx00303b 30090356 PMC6062200

[46] European Commission: Communication on Critical Raw Materials Resilience: Charting a Path towards greater Security and Sustainability.COM/2020/474 final.2020. Reference Source

[47] EuChemS - European Chemical Society: Element Scarcity - EuChemS Periodic Table.2019. Reference Source

[48] Bobba S Carrara S Huisman J : Critical Raw Materials for Strategic Technologies and Sectors in the EU - a Foresight Study. European Commission,2020. 10.2873/58081

[49] European Commission: Study on the EU’s list of critical raw materials - Non-Critical Raw Materials Factsheets.2020. 10.2873/587825

[50] European Commission: Raw Materials Information System (RMIS). Reference Source

[51] Binek A Petrus ML Huber N : Recycling Perovskite Solar Cells to Avoid Lead Waste. *ACS Appl Mater Interfaces.* 2016;8(20):12881–12886. 10.1021/acsami.6b03767 27149009

[52] PV Cycle: Activity Report 2019.2019. Reference Source

[53] Fthenakis V Athias C Blumenthal A : Sustainability evaluation of CdTe PV: An update. *Renew Sustain Energy Rev.* 2020;123:109776. 10.1016/j.rser.2020.109776

[54] Kadro JM Pellet N Giordano F : Proof-of-concept for facile perovskite solar cell recycling. *Energy Environ Sci.* 2016;9(10):3172–3179. 10.1039/C6EE02013E

[55] Kadro JM Hagfeldt A : The End-of-Life of Perovskite PV. *Joule.* 2017;1(1):29–46. 10.1016/j.joule.2017.07.013

[56] Poll CG Nelson GW Pickup DM : Electrochemical recycling of lead from hybrid organic–inorganic perovskites using deep eutectic solvents. *Green Chem.* 2016;18(10):2946–2955. 10.1039/C5GC02734A

[57] Kim BJ Kim DH Kwon SL : Selective dissolution of halide perovskites as a step towards recycling solar cells. *Nat Commun.* 2016;7:11735. 10.1038/ncomms11735 27211006 PMC4879253

[58] Celik I Phillips AB Song Z : Environmental analysis of perovskites and other relevant solar cell technologies in a tandem configuration. *Energy Environ Sci.* 2017;10(9):1874–1884. 10.1039/C7EE01650F

[59] Alberola-Borràs JA Vidal R Juárez-Pérez EJ : Relative impacts of methylammonium lead triiodide perovskite solar cells based on life cycle assessment. *Sol Energy Mater Sol Cells.* 2018;179:169–177. 10.1016/j.solmat.2017.11.008

[60] Chowdhury MS Rahman KS Selvanathan V : Recovery of FTO coated glass substrate *Via* environment-friendly facile recycling perovskite solar cells. *RSC Adv.* 2021;11(24):14534–14541. 10.1039/D1RA00338K 35423997 PMC8697776

[61] Liu FW Biesold G Zhang M : Recycling and recovery of perovskite solar cells. *Mater Today.* 2021;43:185–197. 10.1016/j.mattod.2020.11.024

[62] Yang F Wang S Dai P : Progress in recycling organic-inorganic perovskite solar cells for eco-friendly fabrication. *J Mater Chem A.* 2021;9(5):2612–2627. 10.1039/D0TA07495K

[63] Chhillar P Dhamaniya BP Dutta V : Recycling of Perovskite Films: Route toward Cost-Efficient and Environment-Friendly Perovskite Technology. *ACS Omega.* 2019;4(7):11880–11887. 10.1021/acsomega.9b01053 31460298 PMC6682129

[64] Zhang S Shen L Huang M : Cyclic Utilization of Lead in Carbon-Based Perovskite Solar Cells. *ACS Sustainable Chem Eng.* 2018;6(6):7558–7564. 10.1021/acssuschemeng.8b00314

[65] Wang K Ye T Huang X : One-key-reset” recycling of whole perovskite solar cell. *Matter.* 2021;4(7):2522–2541. 10.1016/j.matt.2021.05.023

[66] Feng X Wang S Guo Q : Dialkylamines Driven Two-Step Recovery of NiO _ *x* _/ITO Substrates for High-Reproducibility Recycling of Perovskite Solar Cells. *J Phys Chem Lett.* 2021;12(19):4735–4741. 10.1021/acs.jpclett.1c00735 33983026

[67] Feng X Guo Q Xiu J : Close-loop recycling of perovskite solar cells through dissolution-recrystallization of perovskite by butylamine. *Cell Rep Phys Sci.* 2021;2(2):100341. 10.1016/j.xcrp.2021.100341

[68] Li MH Yang YS Wang KC : Robust and Recyclable Substrate Template with an Ultrathin Nanoporous Counter Electrode for Organic-Hole-Conductor-Free Monolithic Perovskite Solar Cells. *ACS Appl Mater Interfaces.* 2017;9(48):41845–41854. 10.1021/acsami.7b12367 29134795

[69] Valero A Valero A Calvo G : Material bottlenecks in the future development of green technologies. *Renew Sustain Energy Rev.* 2018;93:178–200. 10.1016/j.rser.2018.05.041

[70] European Commission: Study on the EU’s list of critical raw materials - Critical Raw Materials Factsheets.2020. 10.2873/92480

[71] Nuss P Eckelman MJ : Life cycle assessment of metals: A scientific synthesis. *PLoS One.* 2014;9(7):e101298. 10.1371/journal.pone.0101298 24999810 PMC4085040

[72] Rosenbaum RK Huijbregts MAJ Henderson AD : USEtox human exposure and toxicity factors for comparative assessment of toxic emissions in life cycle analysis: Sensitivity to key chemical properties. *Int J Life Cycle Assess.* 2011;16:710–727. 10.1007/s11367-011-0316-4

[73] European Commission: Commission Recommendation of 9 April 2013 on the Use of Common Methods to Measure and Communicate the Life Cycle Environmental Performance of Products and Organisations. *Annex II.* 2013. Reference Source

[74] European Commission: Final Building the Single Market for Green Products Facilitating Better Information on the Environmental Performance of Products and Organisations.2013. Reference Source

[75] Egorova KS Ananikov VP : Toxicity of Metal Compounds: Knowledge and Myths. *Organometallics.* 2017;36(21):4071–4090. 10.1021/acs.organomet.7b00605

[76] Alberola-Borràs JA Baker JA De Rossi F : Perovskite Photovoltaic Modules: Life Cycle Assessment of Pre-industrial Production Process. *iScience.* 2018;9:542–551. 10.1016/j.isci.2018.10.020 30448247 PMC6286418

[77] Li X Zhang F He H : On-device lead sequestration for perovskite solar cells. *Nature.* 2020;578(7796):555–558. 10.1038/s41586-020-2001-x 32076266

[78] Conings B Babayigit A Boyen HG : Fire safety of lead halide perovskite photovoltaics. *ACS Energy Lett.* 2019;4(4):873–878. 10.1021/acsenergylett.9b00546

[79] Jiang Y Qiu L Juarez-Perez EJ : Reduction of lead leakage from damaged lead halide perovskite solar modules using self-healing polymer-based encapsulation. *Nat Energy.* 2019;4:585–593. 10.1038/s41560-019-0406-2

[80] Chen S Deng Y Xiao X : Preventing lead leakage with built-in resin layers for sustainable perovskite solar cells. *Nat Sustain.* 2021;4:636–643. 10.1038/s41893-021-00701-x

