# Supplementary material for: The critical issue of using lead for sustainable massive production of perovskite solar cells: a review of relevant literature
Source: Open Res Eur. 2021 Oct 28;1:44. Originally published 2021 Apr 23. [Version 2] doi: 10.12688/openreseurope.13428.2 (PMC10445902; doi:10.12688/openreseurope.13428.2)
Supplement: Peer review history

# Peer review: Reviewer response for version 1

**DOI:** 10.21956/openreseurope.14498.r27395

**Published:** 2021-09-24

**Authors:** Matthew Davies, Rhys Charles

**Affiliations:** 1 Specific IKC, Materials Research Centre, College of Engineering, Swansea University, Swansea, UK; 2 Applied Photochemistry Group, SPECIFIC, Materials Science and Engineering, Swansea University, Swansea, UK

**General Comments**

 This paper is a very well put together manuscript which meticulously captures the current thoughts on Pb use in perovskite solar cells and will benefit the field. We have provided minor comments/corrections that may be useful. It is well balanced and discusses the benefits and drawbacks of the use of lead in a logical and comprehensive fashion.

**Introduction**

*Statement in paper: “Renewable energy is unquestionably important to ensure a sustainable society, in which both citizens and industries can benefit and develop while respecting the replenishing rate of natural resources.”*

 Whilst the reviewers of course agree with this statement it may be worth noting that renewable energy technologies are not inherently sustainable (but are considerably much better than fossil fuel based alternatives) from an environmental perspective unless sustainability and end-of-life issues are mitigated/planned/designed for. This is mentioned later in the introduction as a challenge for PSCs but it is of course a challenge for all PV technology.

*Statement in paper: “* *At the same time, life cycle assessment (LCA) methodology has been applied to several PSC configurations to evaluate the eco-profiles of the technology ^25–37 ^and understand better which could be the relevant environmental hotspots along the whole PSC life cycle in addition to those related to the use of Pb-based compounds.”*

 This is a comprehensive list of PSC LCA studies, and the conclusions actually point to a number of differing environmental hotspots. A recent conclusion worth noting from Tian *et al* LCA shows us that the use of functionalised glass substrates and precious metals in electrodes in particular are in most environmental impact categories the major contributors owing to the high impacts embedded in these materials form their production. Conclusions show these impacts can be significantly reduced through a circular economy approach to substitute precious metals and capture the glass for reuse in new modules. The embedded impacts in materials are higher than those of the production process itself, although where thermal processes for materials deposition and curing are used, this incurs the majority of the process impact. These are all things that really need to be addressed to make this technology truly sustainable, and actually differ from the conclusions of many other LCAs that have looked predominantly at lab scale fabrication and extrapolated. In light of this we would recommend adding Tian, X., Stranks, S.D., You, F., 2021. Life cycle assessment of recycling strategies for perovskite photovoltaic modules. Nat. Sustain. 1–9. https://doi.org/10.1038/s41893-021-00737-z

 Another relevant reference, although not LCA, to add may be

 Kadro, J.M., Hagfeldt, A., 2017. The End-of-Life of Perovskite PV. Joule 1, 29–46. https://doi.org/10.1016/j.joule.2017.07.013

 They make comment on the issue of supply bottlenecks for solar glass substrates and a number of the other issues you touch on in this paper.

*Statement in paper: *“Regarding PV applications, although the RoHS2 Directive excludes solar panels from the restrictions, as a precaution PSCs should respect 0.1% Pb content as the maximum concentration value tolerated by weight in “homogeneous material”.”

 This is true, however the exemption will not apply if the PV is product integrated, and so respecting the RoHS limit will unlock this important application also. This could be very important in the context of powering IoT devises, many of which will be important to achieve the sorts of energy efficiency measures required to meet emissions targets also.

*Reference 22* 

 We are unsure what reference this makes to consideration of the leakage of Pb issue?

*Statement in paper: *“Next, we present the most recent mitigation and encapsulation techniques developed and published recently, and finally, we outline the main end-of-life concerns and potential future recycling techniques.”

 No need to say most recent and recently in same sentence.

**Availability, viable alternatives and recyclability**

*Statement in paper: “In this regard, several authors have already explored and investigated the feasibility of the PSC recycling process ^52,53^, highlighting the high recycling rate of Pb ^54^ and the potential recovery of most to all of the PSC components ^55–57^.”*

 With regard to references 55-57, there is a significant body of literature with including attempts to recycle PSCs which does show recovery of most to all of the cell components is possible in addition to the reference cited here. I would suggest that addition of additional references here so to cover  the breadth of this knowledge to the same extent that the authors have done (commendably) for LCA earlier in the paper would strengthen the paper.

*Statement in paper: “Although these processes have been demonstrated at a laboratory scale and need to be scaled up, the prospect of manufacturing PSCs with recovered materials, avoiding efficiency losses, has been already documented ^49^.”*

 Binek’s paper [49] shows the potential for reusing the FTO substrate with no efficiency loss, however this is the only component of the cell (although very important from LCA and cost point of view) that was reused without loss in device efficiency. The paper specifically states results lower PCE in devices made from recovered perovskite precursor solutions. Although we agree with the statement that using recovered materials without efficiency loss has been demonstrated, the reference only shows this to be the case for substrates. I think a quick review as suggested in the comment above will show more research which has managed to reuse more cell components including the perovskite and ETLs without loss in efficiency which you could add here to support this statement.

*Statement in paper*: *“Analysing the possible replacement of Pb with other metals, some critical issues need to be addressed. As reported above, metals identified and tested as a viable alternative for metal-based perovskite in PSCs are Sn, Ge, Bi, Sb and In38. Figure 2 shows that most of these metals’ natural availability is lower than Pb.”*

 Although it is true that the natural availability of these metals is lower than Pb, that is not necessarily what Figure 2 shows specifically. As it is a criticality analysis, it takes into account other factors within supply risk in addition to natural abundance and production including geopolitical factors, global supply and demand, supply concentration, and rate of import in the EU all of which may affect to the materials for the EU, but when combined do not necessarily reflect the natural availability picture alone. The authors go on to state that production rate may be an issue (which implies that natural abundance itself may not be the nature of the bottleneck but the rate at which they can be delivered to the global market), although it is expected that total demand from all sectors for some of these metals will exceed known reserves in coming decades. There are some papers that have examined this, including:

 Valero, Alicia, Valero, Antonio, Calvo, G., Ortego, A., 2018. Material bottlenecks in the future development of green technologies. Renew. Sustain. Energy Rev. 93, 178–200. https://doi.org/10.1016/j.rser.2018.05.041

**LCA for environmental assessment of Pb in PSCs**

*Statement in paper*: *“The main critical raw materials employed for PSC production are gold (used as back contact), the conductive solar glass, and the electron transport material (ETM) due to the organic compounds consumed during the synthesis.”*

 From the references stated in the previous paragraph, it is not clear to me what organic CRM compounds are used in the synthesis of ETLs and how these are critical, or even how they contribute to the lifecycle impacts outlined in the studies mentioned in the previous paragraphs. It may be a confusion in the language. The previous paragraph states that this paragraph summarises the environmental hotspots, which certainly gold and the substrates are, but now we seem to be talking about critical raw materials. Perhaps it is confusing to talk previously in the paper about CRMs and now perhaps we are talking about materials critical to overall environmental impacts, and if so, we would suggest care with the use of the term critical here, and perhaps use the term ‘significant’ instead. Also, it could be made clear what organic materials used in ETM synthesis are significant? From reading the authors previous paper and other references, curing of the ETMs is significant due to energy demand of these thermal processes, and perhaps volatilisation of solvents in this process may also be significant to environmental impacts? Clarification of this would also be helpful to the reader, because the ETMs themselves are generally inorganic oxides or is this specific to all organic ETM?

**Encapsulation as a viable solution to facilitate commercialization**

 While of course not all topics can be covered it may be worth noting/commenting on the work on resin layers to absorb lead/prevent leakage to the environment? e.g., Chen, S., Deng, Y., Xiao, X. *et al.* Preventing lead leakage with built-in resin layers for sustainable perovskite solar cells. *Nat Sustain* 4, 636–643 (2021) https://doi.org/10.1038/s41893-021-00701-x This could be in addition to encapsulation if needed.

Is the review written in accessible language?

Yes

Are all factual statements correct and adequately supported by citations?

Yes

Are the conclusions drawn appropriate in the context of the current research literature?

Yes

Is the topic of the review discussed comprehensively in the context of the current literature?

Yes

Reviewer Expertise:

Perovskite PV, applied photochemistry, circular economy, sustainability and critical raw materials

We confirm that we have read this submission and believe that we have an appropriate level of expertise to confirm that it is of an acceptable scientific standard.

## References

[1] : Life cycle assessment of recycling strategies for perovskite photovoltaic modules. *Nature Sustainability* .2021;4(9) : 10.1038/s41893-021-00737-z 821-829 10.1038/s41893-021-00737-z

[2] : The End-of-Life of Perovskite PV. *Joule* .2017;1(1) : 10.1016/j.joule.2017.07.013 29-46 10.1016/j.joule.2017.07.013

[3] : Material bottlenecks in the future development of green technologies. *Renewable and Sustainable Energy Reviews* .2018;93: 10.1016/j.rser.2018.05.041 178-200 10.1016/j.rser.2018.05.041

[4] : Preventing lead leakage with built-in resin layers for sustainable perovskite solar cells. *Nature Sustainability* .2021;4(7) : 10.1038/s41893-021-00701-x 636-643 10.1038/s41893-021-00701-x

# Response

**Published:** 2021-10-15

**Authors:** Riccardo Basosi

**Affiliations:** University of Siena, Italy

**General Comments**

 This paper is a very well put together manuscript which meticulously captures the current thoughts on Pb use in perovskite solar cells and will benefit the field. We have provided minor comments/corrections that may be useful. It is well balanced and discusses the benefits and drawbacks of the use of lead in a logical and comprehensive fashion.

- ***Response: *** We express our sincere thanks for the efforts of the Reviewers in the evaluation of our manuscript. We are very grateful for the positive and constructive comments, which have been very helpful in preparing an improved revised version of the manuscript that we hope now fully meets the criteria for indexing.

**Introduction**

*Statement in paper: “Renewable energy is unquestionably important to ensure a sustainable society, in which both citizens and industries can benefit and develop while respecting the replenishing rate of natural resources.”*

 Whilst the reviewers of course agree with this statement it may be worth noting that renewable energy technologies are not inherently sustainable (but are considerably much better than fossil fuel based alternatives) from an environmental perspective unless sustainability and end-of-life issues are mitigated/planned/designed for. This is mentioned later in the introduction as a challenge for PSCs but it is of course a challenge for all PV technology.

- ***Response: *** We fully agree with the Reviewers and we have implemented the text accordingly.

*Statement in paper: “At the same time, life cycle assessment (LCA) methodology has been applied to several PSC configurations to evaluate the eco-profiles of the technology ^25–37 ^and understand better which could be the relevant environmental hotspots along the whole PSC life cycle in addition to those related to the use of Pb-based compounds.”*

 This is a comprehensive list of PSC LCA studies, and the conclusions actually point to a number of differing environmental hotspots. A recent conclusion worth noting from Tian *et al* LCA shows us that the use of functionalised glass substrates and precious metals in electrodes in particular are in most environmental impact categories the major contributors owing to the high impacts embedded in these materials form their production. Conclusions show these impacts can be significantly reduced through a circular economy approach to substitute precious metals and capture the glass for reuse in new modules. The embedded impacts in materials are higher than those of the production process itself, although where thermal processes for materials deposition and curing are used, this incurs the majority of the process impact. These are all things that really need to be addressed to make this technology truly sustainable, and actually differ from the conclusions of many other LCAs that have looked predominantly at lab scale fabrication and extrapolated. In light of this we would recommend adding Tian, X., Stranks, S.D., You, F., 2021. Life cycle assessment of recycling strategies for perovskite photovoltaic modules. Nat. Sustain. 1–9. https://doi.org/10.1038/s41893-021-00737-z

 Another relevant reference, although not LCA, to add may be

 Kadro, J.M., Hagfeldt, A., 2017. The End-of-Life of Perovskite PV. Joule 1, 29–46. https://doi.org/10.1016/j.joule.2017.07.013

 They make comment on the issue of supply bottlenecks for solar glass substrates and a number of the other issues you touch on in this paper.

- ***Response: *** We agree with the Reviewers’ suggestion and we have implemented the bibliographic list with the work by Tian and co-authors. We have added also a Nature Sustainability “News and Views” article referred to that work. Concerning the work by Kadro and Hagfeldt, this paper has already been cited in the manuscript in section “Availability, viable alternatives and recyclability”.

*Statement in paper: *“Regarding PV applications, although the RoHS2 Directive excludes solar panels from the restrictions, as a precaution PSCs should respect 0.1% Pb content as the maximum concentration value tolerated by weight in “homogeneous material”.”

 This is true, however the exemption will not apply if the PV is product integrated, and so respecting the RoHS limit will unlock this important application also. This could be very important in the context of powering IoT devises, many of which will be important to achieve the sorts of energy efficiency measures required to meet emissions targets also.

- ***Response: *** We fully agree with the Reviewers and a reference to this issue has been made in the manuscript

*Reference 22* - We are unsure what reference this makes to consideration of the leakage of Pb issue?

- ***Response:*** We thank the Reviewers for having highlighted this typo. We have removed such reference from the text.

*Statement in paper: *“Next, we present the most recent mitigation and encapsulation techniques developed and published recently, and finally, we outline the main end-of-life concerns and potential future recycling techniques.”

 No need to say most recent and recently in same sentence.

- ***Response:*** We thank the Reviewers for having highlighted this typo. We have corrected the text accordingly.

**Availability, viable alternatives and recyclability**

*Statement in paper: “In this regard, several authors have already explored and investigated the feasibility of the PSC recycling process ^52,53^, highlighting the high recycling rate of Pb ^54^ and the potential recovery of most to all of the PSC components ^55–57^.”*

 With regard to references 55-57, there is a significant body of literature with including attempts to recycle PSCs which does show recovery of most to all of the cell components is possible in addition to the reference cited here. I would suggest that addition of additional references here so to cover  the breadth of this knowledge to the same extent that the authors have done (commendably) for LCA earlier in the paper would strengthen the paper.

- ***Response:*** According to the Reviewers’ suggestion we have added further bibliographic references to support the statement.

*Statement in paper: “Although these processes have been demonstrated at a laboratory scale and need to be scaled up, the prospect of manufacturing PSCs with recovered materials, avoiding efficiency losses, has been already documented ^49^.”*

 Binek’s paper [49] shows the potential for reusing the FTO substrate with no efficiency loss, however this is the only component of the cell (although very important from LCA and cost point of view) that was reused without loss in device efficiency. The paper specifically states results lower PCE in devices made from recovered perovskite precursor solutions. Although we agree with the statement that using recovered materials without efficiency loss has been demonstrated, the reference only shows this to be the case for substrates. I think a quick review as suggested in the comment above will show more research which has managed to reuse more cell components including the perovskite and ETLs without loss in efficiency which you could add here to support this statement.

- ***Response:*** According to the Reviewers’ suggestion we have added further bibliographic references to support the statement.

*Statement in paper*: *“Analysing the possible replacement of Pb with other metals, some critical issues need to be addressed. As reported above, metals identified and tested as a viable alternative for metal-based perovskite in PSCs are Sn, Ge, Bi, Sb and In38. Figure 2 shows that most of these metals’ natural availability is lower than Pb.”*

 Although it is true that the natural availability of these metals is lower than Pb, that is not necessarily what Figure 2 shows specifically. As it is a criticality analysis, it takes into account other factors within supply risk in addition to natural abundance and production including geopolitical factors, global supply and demand, supply concentration, and rate of import in the EU all of which may affect to the materials for the EU, but when combined do not necessarily reflect the natural availability picture alone. The authors go on to state that production rate may be an issue (which implies that natural abundance itself may not be the nature of the bottleneck but the rate at which they can be delivered to the global market), although it is expected that total demand from all sectors for some of these metals will exceed known reserves in coming decades. There are some papers that have examined this, including:

 Valero, Alicia, Valero, Antonio, Calvo, G., Ortego, A., 2018. Material bottlenecks in the future development of green technologies. Renew. Sustain. Energy Rev. 93, 178–200. https://doi.org/10.1016/j.rser.2018.05.041

- ***Response:*** We thank the Reviewers for having highlighted this point that, actually, created a misunderstanding. We fully agree with Reviewers’ comment, thus we have modified the text erasing the adjective “natural” and we added the suggested reference.

**LCA for environmental assessment of Pb in PSCs**

*Statement in paper*: *“The main critical raw materials employed for PSC production are gold (used as back contact), the conductive solar glass, and the electron transport material (ETM) due to the organic compounds consumed during the synthesis.”*

 From the references stated in the previous paragraph, it is not clear to me what organic CRM compounds are used in the synthesis of ETLs and how these are critical, or even how they contribute to the lifecycle impacts outlined in the studies mentioned in the previous paragraphs. It may be a confusion in the language. The previous paragraph states that this paragraph summarises the environmental hotspots, which certainly gold and the substrates are, but now we seem to be talking about critical raw materials. Perhaps it is confusing to talk previously in the paper about CRMs and now perhaps we are talking about materials critical to overall environmental impacts, and if so, we would suggest care with the use of the term critical here, and perhaps use the term ‘significant’ instead. Also, it could be made clear what organic materials used in ETM synthesis are significant? From reading the authors previous paper and other references, curing of the ETMs is significant due to energy demand of these thermal processes, and perhaps volatilisation of solvents in this process may also be significant to environmental impacts? Clarification of this would also be helpful to the reader, because the ETMs themselves are generally inorganic oxides or is this specific to all organic ETM?

- ***Response:*** We thank the Reviewers for having highlighted this point that, actualy, created a misunderstanding. We have substituted “critical raw materials” with “hotspots in terms of materials” to clarify better this point and implemented the text concerning the environmental load characterizing the ETM production.

**Encapsulation as a viable solution to facilitate commercialization**

 While of course not all topics can be covered it may be worth noting/commenting on the work on resin layers to absorb lead/prevent leakage to the environment? e.g., Chen, S., Deng, Y., Xiao, X. *et al.* Preventing lead leakage with built-in resin layers for sustainable perovskite solar cells. *Nat Sustain* 4, 636–643 (2021) https://doi.org/10.1038/s41893-021-00701-x This could be in addition to encapsulation if needed.

- ***Response***: We thank the Reviewers for the reference to this interesting work that has been added and commented in the manuscript.

# Peer review: Reviewer response for version 1

**DOI:** 10.21956/openreseurope.14498.r26802

**Published:** 2021-06-04

**Authors:** Claudia Barolo, Nicole Mariotti, Matteo Bonomo

**Affiliations:** 1 NIS Interdepartmental Centre and INSTM Reference Centre, Department of Chemistry, Università degli Studi di Torino, Torino, Italy

This is a very clear and well written review on ALL ASPECTS connected with the sustainability and environmental assessment of Pb employed in PSCs. This very useful literature analysis starts from the physico-chemical properties of Pb and its potential risks for humans and the environment. Afterward, the major outcomes of LCA, toxicological and risk assessment on PSCs, are presented focusing on the toxicity-related environmental impacts. Then, the most recent mitigation and encapsulation techniques are discussed and finally the main end-of-life concerns and potential future recycling techniques outlined.

 The topic of this review is timely and has been discussed comprehensively in the context of the current literature. Many factual statements have been reported and adequately supported by correct citations. The language is clear and appropriate. The conclusions fit well in the context of the current research literature.

 The paragraphs Pb in PSCs clearly states how Pb is fundamental for the high performances of the PV device as till now, no viable, effective and compelling alternative has been found. However the analysis of the literature regarding the toxicology issues (well documented) as well as the leakage from perovskite solar modules points out the critical aspects of its use versus other Pb sources. For this reason Table 1 (from ref.19) has been correctly selected and commented. Thus claiming that a substantial reduction of Pb-based compound and GHG emissions could be achieved by replacing conventional energy production technologies with PSCs.

 The discussion on LCA for environmental assessment of Pb in PSCs is very well conducted and the general output clearly suggests that the evaluation of potential toxicity of Pb-based PSCs could be still an open question: more detailed and in-depth LCA studies focusing on the life cycle of Pb-based compounds employed are necessary to evaluate the real sustainability of Pb-based PSCs. Of course the availability of suitable data along the whole value chain (not only materials but also design of the cells, technology, encapsulation, etc.) will offer the possibility to assess the real potentiality and sustainability asset of this innovative technological approach.

**Few really minor remarks:**

**Page 3**

 "In sharp contrast with all the other fuels, renewables have already shown their resilience to the coronavirus pandemic crisis."

- We agree with the authors, but an adequate reference or a more specific explanation in the text could be added.

**Page 4**

 "The highest efficiency recorded for a Pb-free PSC is 9.6%, reached with a Sn-based perovskite…"

- We agree with the sentence however a novel record (10.9%, Chen *et al.* (2020) ^1^) and a recent review on the topic can be useful for the reader (e.g., Nasti & Abate (2020) ^2^).

**Page 6**

 "Nuss *et al.* performed an LCA of metals…"

- Maybe rephrasing as, “Nuss *et al.* performed a cradle-to-gate LCA of metals”, is more informative for the reader

**Page 7**

 "The main critical raw materials"

- Here, the simple use of “critical materials” without “raw” could be more easy and general.

**References**

 Refs: 8 ,9

- Year missing: is that made with the idea to keep the reference updated?

A list of the used abbreviations (even if all of them have been indicated in the text) could help the readers, especially those not completely familiar to the field.

Is the review written in accessible language?

Yes

Are all factual statements correct and adequately supported by citations?

Yes

Are the conclusions drawn appropriate in the context of the current research literature?

Yes

Is the topic of the review discussed comprehensively in the context of the current literature?

Yes

Reviewer Expertise:

Material synthesis and developing of innovative photovoltaics

We confirm that we have read this submission and believe that we have an appropriate level of expertise to confirm that it is of an acceptable scientific standard.

## References

[1] : High-Performance Lead-Free Solar Cells Based on Tin-Halide Perovskite Thin Films Functionalized by a Divalent Organic Cation. *ACS Energy Letters* .2020;5(7) : 10.1021/acsenergylett.0c00888 2223-2230 10.1021/acsenergylett.0c00888

[2] : Tin Halide Perovskite (ASnX3 ) Solar Cells: A Comprehensive Guide toward the Highest Power Conversion Efficiency. *Advanced Energy Materials* .2020;10(13) : 10.1002/aenm.201902467 10.1002/aenm.201902467

# Response

**Published:** 2021-10-15

**Authors:** Riccardo Basosi

**Affiliations:** University of Siena, Italy

We express our sincere thanks for the efforts of the Reviewers in the evaluation of our manuscript. We are very grateful for the positive and constructive comments. We have taken into account all their remarks in preparing an improved revised version of the manuscript that we hope now fully meets the criteria for publication in Open Research Europe.

**Page 3**

 "In sharp contrast with all the other fuels, renewables have already shown their resilience to the coronavirus pandemic crisis." We agree with the authors, but an adequate reference or a more specific explanation in the text could be added.

- ***Response***: We have added a reference to contextualize better this assertion.

**Page 4**

 "The highest efficiency recorded for a Pb-free PSC is 9.6%, reached with a Sn-based perovskite…"

 We agree with the sentence however a novel record (10.9%, Chen *et al.* (2020) ^1^ ) and a recent review on the topic can be useful for the reader (e.g., Nasti & Abate (2020) ^2^ ).

- ***Response***: We thank the Reviewers for this suggestion and we have implemented the text accordingly.

**Page 6**

 "Nuss *et al.* performed an LCA of metals…"Maybe rephrasing as, “Nuss *et al.* performed a cradle-to-gate LCA of metals”, is more informative for the reader.

- ***Response: *** We have modified the text accordingly.

**Page 7**

 "The main critical raw materials"- Here, the simple use of “critical materials” without “raw” could be more easy and general.

- ***Response: *** We agree with the Reviewers that the term “raw” could have raised some misunderstanding, thus we have changed the expression “main critical raw materials” in “main hotspots in terms of materials”

**References**

 Refs: 8 ,9- Year missing: is that made with the idea to keep the reference updated?

- ***Response*** *: *Yes, our intention is to refer to the NREL charts that are continuously updated A list of the used abbreviations (even if all of them have been indicated in the text) could help the readers, especially those not completely familiar to the field. Actually we have formatted the manuscript according to the article guidelines given by the publishing platform. This is the reason why an abbreviation list is not present in the manuscript.  
